# Niche Competition and Overlapping Area Dynamics of Two Sympatric Ants Jointly Indicate Strong Adaptive and Dispersal Ability of Yellow Crazy Ant (*Anoplolepis gracilipes*)

**DOI:** 10.3390/ani15172633

**Published:** 2025-09-08

**Authors:** Yulin Yuan, Changqi Chen, Ying Zhang, Jinlu Zhang, Zhouyang Liao, Fang Liu, Zachary Y. Huang, Yuan Zhang

**Affiliations:** 1 Key Laboratory of Forest Disaster Warning and Control of Yunnan Province, College of Forestry, Southwest Forestry University, Kunming 650224, China; yulin_yuan@swfu.edu.cn (Y.Y.); chen_cq@swfu.edu.cn (C.C.); zhangying@swfu.edu.cn (Y.Z.); jinlu_zhang@swfu.edu.cn (J.Z.); zhouyang_liao@swfu.edu.cn (Z.L.); 2 College of Forestry, Southwest Forestry University, Kunming 650224, China; 3 Institute of Zoology, Guangdong Academy of Sciences, Guangzhou 510260, China; liufang@giz.gd.cn; 4 Department of Entomology, Michigan State University, East Lansing, MI 48824, USA; bees@msu.edu

**Keywords:** *Anoplolepis gracilipes*, *Oecophylla smaragdina*, climate change, sympatric distribution, niche competition, species distribution model

## Abstract

**Simple Summary:**

Invasive insects, particularly ants, pose a significant threat to biodiversity due to their strong reproductive capabilities and environmental adaptability. Tropical regions are especially vulnerable, as favorable climatic and ecological conditions facilitate the proliferation of invasive ant species. Yellow crazy ants (*Anoplolepis gracilipes*), as one of the IUCN’s top 100 most destructive invasive species, also the first documented chimeric ant species, may exploit this unique genetic trait to survive in adverse environments. Therefore, investigating their interactions with native species is both scientifically meaningful and practically essential. Existing studies and our preliminary observations indicate that yellow crazy ants frequently coexist with weaver ants (*Oecophylla smaragdina*), a dominant native species. This study employed field-controlled experiments and species distribution models to predict the distribution dynamics of two ant species. Our results suggest that the yellow crazy ant has a strong competitive ability in the wild, and the overlapping area between the two species is shrinking. Under future climate change scenarios, yellow crazy ants are projected to expand their range, while weaver ants are expected to decline. Our findings suggest that it is imperative to closely monitor the spread of this invasive ant species.

**Abstract:**

Global climate change, coupled with the escalating severity of species invasions, has profoundly impacted and continues to influence species distribution patterns across multiple spatial scales. The invasive ant species *Anoplolepis gracilipes* (yellow crazy ants) and the dominant species *Oecophylla smaragdina* (weaver ants) share a significant overlapping distribution in tropical Asia and Oceania. The changes in their distribution areas, particularly in the overlapping regions, under future climate change scenarios remain inadequately explored. By integrating field behavioral experiments conducted on two ant species with climate and topographic datasets, we evaluated the extent of overlapping ranges and predicted the future dynamics of both species. Our results show that yellow crazy ants are more efficient at finding food and mobilizing workers, indicating stronger collaborative abilities than weaver ants. Under food and water deprivation conditions, yellow crazy ants exhibit a higher survival rate than weaver ants. Climatic factors exert a greater influence on the potential distribution of yellow crazy ants compared to topographic factors. Regions with consistently high suitability for yellow crazy ants primarily include southern China, Myanmar, India, Thailand, Malaysia, and Australia. The potential distribution area for weaver ants has constricted due to climate change, while that for yellow crazy ants has expanded. Initially, these two ant species had highly overlapping suitable habitats. However, this overlap is projected to diminish under future climate conditions. Mitigating future climate change could substantially reduce the expansion of yellow crazy ants. This discovery underscores the importance of monitoring and managing the dynamic changes in the distribution areas of both invasive and native species against the backdrop of climate change.

## 1. Introduction

Biological invasions are one of the major problems affecting economic trade, agroforestry, ecosystems and biodiversity in the world today [1]. In recent years, due to the rapid development of international trade and tourism industry on a global scale, the introduction risk of invasive species has also risen sharply. The environmental and ecological problems caused by invasive species have also become increasingly severe [2]. A lot of research has shown that invasive species can reduce the species richness and even lead to the extinction of some native species, causing irreversible damages to biodiversity [3]. In recent years, many studies have been carried out on the impact of invasive species on native species, and the results indicate that invasive species may affect native species by virtue of their behavioral and population size superiority, leading to changes in native communities [4,5]. Invasive species have become a major factor in the decline of biodiversity in many regions [6].

Invasive insects, particularly ants, pose a significant threat to biodiversity due to their strong reproductive capacity and adaptability. Researchers are increasingly focusing on the impact of invasive insects on native species, but this area remains insufficiently studied [7,8]. Invasive ants can cause substantial declines in biodiversity by competing directly and indirectly with native ants for resources and habitat space [9,10]. Their competitive advantages include high interspecific aggression, resource monopolization, and strong adaptability to environmental changes [11,12,13,14]. In the tropical area, favorable environmental and climatic conditions promote the colonization, spread, and outbreaks of invasive ants, making this region highly vulnerable to their impact [15]. For instance, yellow crazy ants have reduced ant diversity in the Tokelau Islands [16], killed red crabs on Christmas Island by spraying formic acid, leading to a dramatic population decline [17], and are recognized among the IUCN’s top 100 most destructive invasive species [18,19,20]. These ants aggressively prey on a wide range of organisms, resulting in substantial declines in biodiversity. While research on invasive ants is growing, most studies focus on the fire ant *Solenopsis invicta*, with limited attention given to the yellow crazy ants’ biological and ecological traits [21].

A study published in Science in 2023 revealed an unusual reproductive system in the yellow crazy ant. When sperm carrying the W genome fuses with an egg, the offspring develops into a chimeric male; otherwise, it becomes a worker [22]. This genetic mechanism enables ants to dynamically adjust their population structure. In resource-rich environments, queens can rapidly expand the colony by producing more workers. In contrast, under competitive conditions, the mobility of chimeric males may facilitate gene flow. Chimerism also enhances genetic diversity among workers, which may contribute to the formation of supercolonies. These characteristics likely enhance the ants’ ability to successfully colonize new habitats [22]. While chimerism occasionally occurs in other insect species, it is typically regarded as a developmental anomaly, and the resulting males are often sterile [23]. The yellow crazy ant is the first known species in which chimerism is intrinsic biological feature. Investigating how these ants interact with other species is, therefore, both scientifically significant and ecologically relevant.

According to previous studies and our preliminary investigations, there is a significant overlap in the distribution areas of yellow crazy ants and weaver ants [21]. As a dominant ant species in many tropical regions and one of the earliest reported biological control insects globally, weaver ants play a crucial role in tropical ecosystems [23,24,25,26]. Research has demonstrated that weaver ants can effectively control over 20 pest species on economically important trees such as mango, citrus, cacao, cashew nuts, and coconuts [27,28,29]. For instance, the presence of weaver ants has been shown to reduce damage caused by *Metisa plana* to oil palms in Asia, thereby enhancing palm oil production [30]. However, with the intensification of climate change and biological invasions, weaver ants, a dominant species in many tropical regions, may be experiencing population declines. In the Xishuangbanna area of Yunnan, located at the China–Thailand border and characterized by a typical tropical rainforest climate, we observed through five years of field studies that the distribution of yellow crazy ants increased in some experimental plots, while the population of weaver ants showed signs of decline (personal observations).

Global climate change, biological invasions, land use changes, and increased human activities threaten insect diversity and distribution, altering biological interactions [31]. Studies show that climate change affects the seasonal activity, species richness, and geographic ranges of certain ant species [32,33]. Given the overlapping distributions of yellow crazy ants and weaver ants, ecological niche competition between them is likely. Ants can enhance reproductive success and foraging efficiency by adapting to biotic and abiotic changes, expanding their ecological niches and increasing population sizes [34,35]. The Fifth Assessment Report of the Intergovernmental Panel on Climate Change (IPCC AR5) indicates that global climate change is significantly altering the climate environment. The global mean temperature from 2001 to 2020 was 0.99 °C higher than the average from 1850 to 1900 [36]. Studies suggest that invasive species may adapt their ecological niches through evolution, potentially expanding into new regions. Future climate change could, thus, impact species distribution more profoundly [37]. Controlled indoor experiments show that factors like temperature, humidity, altered precipitation patterns, and elevated CO_2_ levels can influence ant reproduction, survival, and growth [38,39,40,41].

As tropical insects, weaver ants are highly sensitive to environmental changes [42]. While climate change may expand invasive species’ distributions, its impact on yellow crazy ants and weaver ants remains unclear. This study investigates the foraging behavior and potential distribution dynamics of dominant and invasive ant species by field experiments, modeling predictions, and assessments of niche overlap between the two species under global climate change scenarios. Our findings enhance understanding of their ecological competitiveness and predict future distributions, providing a scientific basis for preventing the spread of yellow crazy ants and protecting weaver ants, as well as offering insights into interactions between invasive and native species.

## 2. Materials and Methods

### 2.1. Study Area and Experimental Insect

The study area encompasses the distribution regions of two ant species, as well as the field experiment sites. The field competition experiment was conducted in the Xishuangbanna Tropical Botanical Garden, Chinese Academy of Sciences, situated on the northern fringe of the Indo-Malayan tropical rainforest region (20°4′ N, 101°25′ E), bordering Myanmar and Laos. This region experiences a tropical monsoon climate at an elevation of 570 m, with an average annual temperature of 21.4 °C, with no frost throughout the year [43]. The data collection area encompassed the sympatric distribution region of two ant species, primarily located between the Tropic of Cancer and the Tropic of Capricorn. This region includes southern China, the Indo-Malay zone, and northern Australia. The area is characterized by its rich biological resources, serving as a habitat for numerous rare and endangered species, and encompassing many of the world’s biodiversity hotspots. However, it is also one of the regions most affected by biological invasions [44].

Both weaver ants (*Oecophylla smaragdina*) and yellow crazy ants (*Anoplolepis gracilipes*) belong to the family Formicidae in Hymenoptera. The ants used in the controlled experiment were collected from Xishuangbanna, Yunnan, China. To minimize hyperactivity due to the stress of collection, the ants were acclimatized in a dark environment for 24 h prior to the experiments. The collected ants were placed in collection tubes plugged with moist cotton. They were fed daily with a 20% sugar water solution and maintained in an incubator set at 25 °C with 80% humidity.

### 2.2. Comparison of Competitive Ability for Food Resources in the Wild Between Both Species

The experiment was conducted from December 2019 to November 2020. Plastic cups (8 cm in diameter) were modified by cutting to a depth of 1 cm to facilitate ant entry. For the baiting experiment, small pieces of apple, sausage, and 10 mL of 50% honey water were placed separately at the bottom of cup. Three cups were placed near the base of each tree where two ant species had been previously observed. Each food item was placed ~2 m away from the base of the trees and ~2 m apart from one another. Three researchers continuously monitored and recorded the arrival time of the first ant and counted the number of ants every 30 min for each bait. Workers were counted every 30 min over a 2 h observation period, with the highest count taken as the maximum number of workers visited. The field experiment was conducted from 9:00 am to 11:00 am each day. A total of 5 trees were used, with each tree spaced at least 200 m apart, with a 5-day interval between each session. To ensure variability and representativeness, we collected ants from different colonies in each experimental session. Thus, there were a total of five sessions, with 25 replications conducted per treatment condition. If it rained on the scheduled day, the experiment was postponed by one day. This experiment was carried out in the natural setting.

### 2.3. Study on Aggression Behavior and Ability for Both Species

We collected both species for aggression experiments. Ants of the same species were collected from the same colony for each experimental session to minimize intraspecific aggression, while ants used on different experimental days were sourced from different colonies. Workers with similar body sizes were selected and individually housed in clean insect collection tubes (20 mm diameter). The experimental ants were carefully transferred into the Petri dish using a soft brush. Three experimental combinations were tested: one weaver ant versus one crazy yellow ant (Combination 1), one weaver ant versus five crazy yellow ants (Combination 2), and one crazy yellow ant versus five weaver ants (Combination 3). Each experimental design was replicated 30 times, and divided into three batches, with ten replicates per batch. Each ant was used only once in the aggression behavior experiment.

We continuously observed the ants from the moment they were introduced into the petri dish, and it was recorded whether any aggression occurred. If no aggression was observed within 30 min, the interaction was classified as no contact. When aggression did occur, the intensity of the attack was categorized into four levels: level I, brief antennal contact (<1 s); level II, prolonged antennal contact (>1 s); level III, lifting each other’s abdomen, opening mandibles, or rapidly climbing onto the other’s back; and level IV, sustained entanglement while attacking or one ant forcefully biting the other with its mandibles [45,46]. After a 30 min observation period, the number of dead individuals and mortality rates for each species were recorded. Ants were classified as dead if they exhibited no movement when touched with forceps, or if their bodies were severely contorted and unable to maintain an upright position.

### 2.4. Hunger and Thirst Tolerance Ability for Both Species

To evaluate the hunger and thirst tolerance abilities of both species, neither food nor water was provided during the experimental period. Medium-sized worker ants of yellow crazy ants and weaver ants were collected while foraging outside the nest. To minimize potential interference, each ant was placed in a separate sterile tube (20 mm diameter) with a cotton plug to ensure adequate air permeability. Five rounds of experiments were conducted for each species, using ants from the same colony within each round, totaling five distinct colonies. Each experiment comprised ten replicates, resulting in a total of 50 replicates. To ensure environmental consistency, tubes containing ants were placed in a climatic chamber (Saifu PRX-350C, Shanghai, China) maintained at 25 °C, with a 12L:12D photoperiod, and 80% relative humidity. Ant survival was assessed daily at 8:00, 16:00, and 24:00; ants that failed to respond to tactile stimulation with tweezers were recorded as dead. The experiment continued until all ants died.

### 2.5. Occurrence Records for Both Species

**Figure 1:**
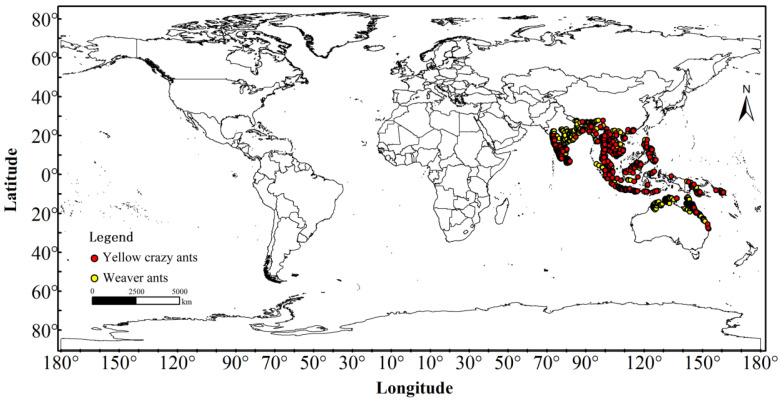
Distribution data of yellow crazy ants (*Anoplolepis gracilipes*) and weaver ants (*Oecophylla smaragdina*) in the sympatric range.

The distribution data for both species were sourced from field surveys, the Global Biodiversity Information Facility (GBIF) database (https://www.gbif.org). GBIF Occurrence Download, available online: (DOI link: 10.15468/dl.wvcv9g) (accessed on 28 June 2023) for *Anoplolepis gracilipes*; (DOI link: 10.15468/dl.z26mdx) (accessed on 28 June 2023) for *Oecophylla smaragdina*. And a comprehensive review of global published literature. Initial filtering was conducted using ENMTools version 1.1.1 in R Studio (version 4.2.2) to eliminate duplicate records and entries with missing geographic coordinates [47,48]. Subsequently, SDMToolbox in ArcGIS 10.8 was employed to remove duplicate records within a 10 km radius [49], ensuring that only one data point was retained per grid cell to prevent model overfitting and enhance model performance [50]. Ultimately, 470 distribution records for yellow crazy ants and 1179 records for weaver ants were retained (Figure 1), which were then converted into CSV format for further analysis.

### 2.6. Environmental Variables

To examine the effects of global climate change on the spatial distribution patterns of yellow crazy ants and weaver ants, we sourced data from WorldClim version 2.1 (https://www.worldclim.org/), which includes 19 standard bioclimatic variables, 1 topographic variable, and projections from the Community Climate System Model version 4 (CCSM4) [51]. For historical climate data, we utilized datasets for the Last Glacial Maximum (LGM) and the Mid-Holocene (MH) periods at a spatial resolution of 30″ (approximately 1 km × 1 km). The LGM represents the most recent period of extreme cold, characterized by significantly colder and drier conditions compared to the present climate. In contrast, the MH period reflects a more recent warm and humid phase, marked by warmer and wetter conditions than those observed today [52]. For contemporary data, we obtained 19 standard bioclimatic variables (averages for 1970–2000) along with altitude data at a spatial resolution of 30″ (approximately 1 km × 1 km). For future climate projections, we acquired data for two representative concentration pathway scenarios (RCP2.6 and RCP8.5) for both 2050 and 2070, covering all aforementioned bioclimatic variables at the same spatial resolution.

The altitude raster files were transformed into slope direction and slope gradient raster files using the 3D Analyst toolbox in ArcGIS version 10.8. Environmental variables were selected based on their significant influence on species distribution and the high inter-correlation among them [53]. To remove highly correlated variables (Pearson correlation coefficient > 0.8), we employed the ENMTools package (version 1.1.0) in RStudio [48]. We selected biologically relevant variables associated with the insect lifespan and prioritized factors that have a significant influence on species distribution (Table 1).

### 2.7. Model Construction and Evaluation

In the MaxEnt model, the distribution data and 12 key environmental factors were input to simulate the historical, current, and future potential habitat areas of the two ant species. To optimize the model, we employed a regularization multiplier (RM) ranging from 0.5 to 4 in increments of 0.5. We evaluated six feature combinations (FC): Linear (L), Linear Quadratic (LQ), Hinge (H), Linear Quadratic Hinge (LQH), Linear Quadratic Hinge Product (LQHP), and Linear Quadratic Hinge Product Threshold (LQHPT). The adjusted AICc values derived from feature combinations tested using the block method in the ENMeval package were used to select the parameter combination with the lowest adjusted AICc value for model simulation [54]. During the simulation, 75% of the distribution point data were randomly selected as the training set to construct the model, while the remaining 25% served as the test set to validate the results. The model was repeated 10 times to ensure stability, and settings included running Jackknife and response curve analyses to evaluate the environmental variables. All other parameters were set to default [55].

The contribution rates of 12 environmental variables were analyzed using the Jackknife method within the MaxEnt model framework. The receiver operating characteristic (ROC) curve analysis was utilized to assess the accuracy of intrusion risk predictions [56]. A threshold of 10% training presence was set to delineate suitable and unsuitable habitats for both species. Subsequently, the natural break classification method in ArcGIS was employed to refine the threshold for identifying suitable areas [57]. To visually illustrate changes in suitable habitats over different periods and under varying conditions, the spatial statistics tool in ArcGIS version 10.8 was used to calculate and display shifts in the geometric centroid positions within potentially suitable areas [50]. Finally, area statistics were performed on the suitable regions to compare the changes in spatial distribution patterns of both species under past, current, and future climatic scenarios.

### 2.8. Spatial Niche Overlap Analysis

Based on the “species presence probability (0–1)” generated by the MaxEnt model and integrated with the actual field distribution records of the target species, the SDMToolbox in ArcGIS was employed to identify grid cells with a presence probability ≥ 0.5 as the “potential suitable habitat”. The spatial niche overlap between the two species was quantified using ENMTools version 1.1.1 within R4.3.3. The degree of spatial niche overlap was assessed through Schoener’s D (*D*) and Hellinger’s distance I (*I*), both metrics ranging from 0 to 1. Higher values indicate a greater degree of spatial niche overlap [58]. The predicted distributions for both species were subsequently mapped into distinct regions using the overlap analysis tool in ArcGIS version 10.8.

$$D\left(P_{O},\;P_{A}\right)=1-\frac{1}{2}\sum_{i}\left|P_{O,i}\right.-\left.P_{A,i}\right|$$

$$I\left(P_{O},\;P_{A}\right)=1-\frac{1}{2}\sqrt{\sum_{i}{\left(\sqrt{P_{O,i}}-\sqrt{P_{A,i}}\right)}^{2}}$$

*P*_*O*,*i*_ and *P*_*A*,*i*_ represent the normalized habitat suitability scores for weaver ants and yellow crazy ants, respectively, for the *i*th raster cell in the MaxEnt model results, and the scores were output as [0,1].

### 2.9. Data Analyses

Data organization was performed using Microsoft Excel 2010, and statistical analysis was conducted using SPSS Statistics (Version 27.0). The *t*-test was employed to compare bait detection time and worker numbers between the two ant species. Chi-square tests were used to examine the differences in aggression behaviors among various ant combinations. Fisher’s exact test was applied to analyze mortality rates during fights, while time series and log-rank tests were utilized to compare the survival rate of the two species, and ggplot2 of R(4.5.0) was used to plot survival curves.

## 3. Results

### 3.1. Food Resource Competitive Ability of Both Species

The observation results indicate that a container with a depth of 1 cm does not present a barrier to foraging activities for either of the two ant species. Among the three different baits used in the experiment, yellow crazy ants showed a significantly higher efficiency for apples (*t* = 9.26, *p* < 0.01) and honey (*t* = 4.81, *p* < 0.01) compared to weaver ants. However, there was no significant difference in the time spent searching for sausage between the two species (*t* = 0.84, *p* > 0.05). Variations were also observed in the maximum number of worker ants recruited by bait type: yellow crazy ants attracted significantly more workers than weaver ants when exposed to apples (*t* = 0.84, *p* < 0.01), honey (*t* = 10.38, *p* < 0.01), but the result of sausages was different (*t* = 4.80, *p* < 0.01) (Table 2).

### 3.2. Comparison of the Aggression Abilities of Both Species

**Figure 2:**
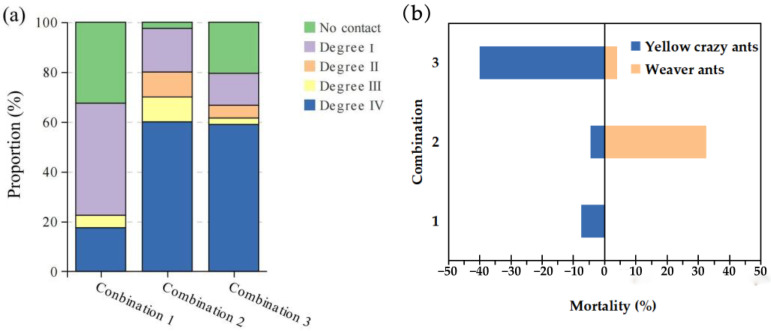
Aggression intensity (**a**) and mortality (**b**) in different combinations of both species. Note: Combination 1: yellow crazy ants vs. weaver ants = 1:1; Combination 2: yellow crazy ants vs. weaver ants = 5:1; Combination 3: yellow crazy ants vs. weaver ants = 1:5.

When the ratio of yellow crazy ants vs. weaver ants was 1:1, no contact and level I aggression accounted for 77.5%. However, when the ratios were 1:5 or 5:1, the intensity of aggression increased markedly, with levels III and IV attacks comprising 70% and 60%, respectively. The Chi-square test revealed significant differences in aggressive behavior across these three ratios (χ^2^ = 890.61, *p* < 0.001) (Figure 2a).

There were also significant differences in mortality rates among different combinations. Within half an hour, when yellow crazy ants and weaver ants were in a 1:1 ratio, the fatality rates were 7.5% and 0%, respectively (*p* < 0.001). At a 5:1 ratio, the mortality rates were 4.5% for yellow crazy ants and 32.5% for weaver ants. Conversely, at a 1:5 ratio, the mortality rates were 40.0% for yellow crazy ants and 4.0% for weaver ants (*p* < 0.001) (Figure 2b).

### 3.3. Survival Time of Workers in the Hunger and Thirst State

**Figure 3:**
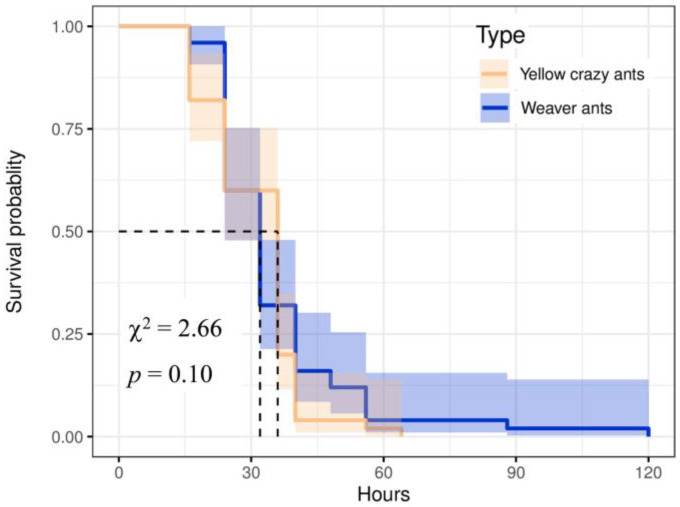
Survivorship curve of yellow crazy ants and weaver ant workers under hunger and thirst state.

The log-rank test was conducted to analyze the survival curve, revealing no significant difference in the survival situation between 2 species (χ^2^ = 2.66, *p* = 0.10). However, yellow crazy ants exhibited a prolonged survival time of up to 120 h without access to water and food compared to weaver ants’ maximum survival time of 64 h; this demonstrated greater intraspecific variation (Figure 3).

### 3.4. Model Performance and Environmental Variable Importance

The reliability of the optimized MaxEnt model was evaluated through ROC curve analysis. Using 12 environmental variables, the ENMeval data package produced mean AUC values of 0.966 for yellow crazy ants and 0.938 for weaver ants. These high AUC values substantiate the model’s robust accuracy in predicting suitable habitats for both species. Based on the contribution percentages and importance rankings derived from the MaxEnt model, bio1, bio2, bio3, bio7, and bio13 were identified as the key environmental variables influencing the distribution of both species (Table 3).

The analysis of optimal climatic ranges for two ant species revealed that the differences in suitable climatic factors between the species were not obvious. The environmental variables derived from the model indicate that yellow crazy ants exhibit a broader tolerance range for annual mean temperature and isothermality compared to weaver ants (Table 4).

### 3.5. Suitable Habitat Dynamics and Geometric Centroid for Two Ant Species

**Figure 4:**
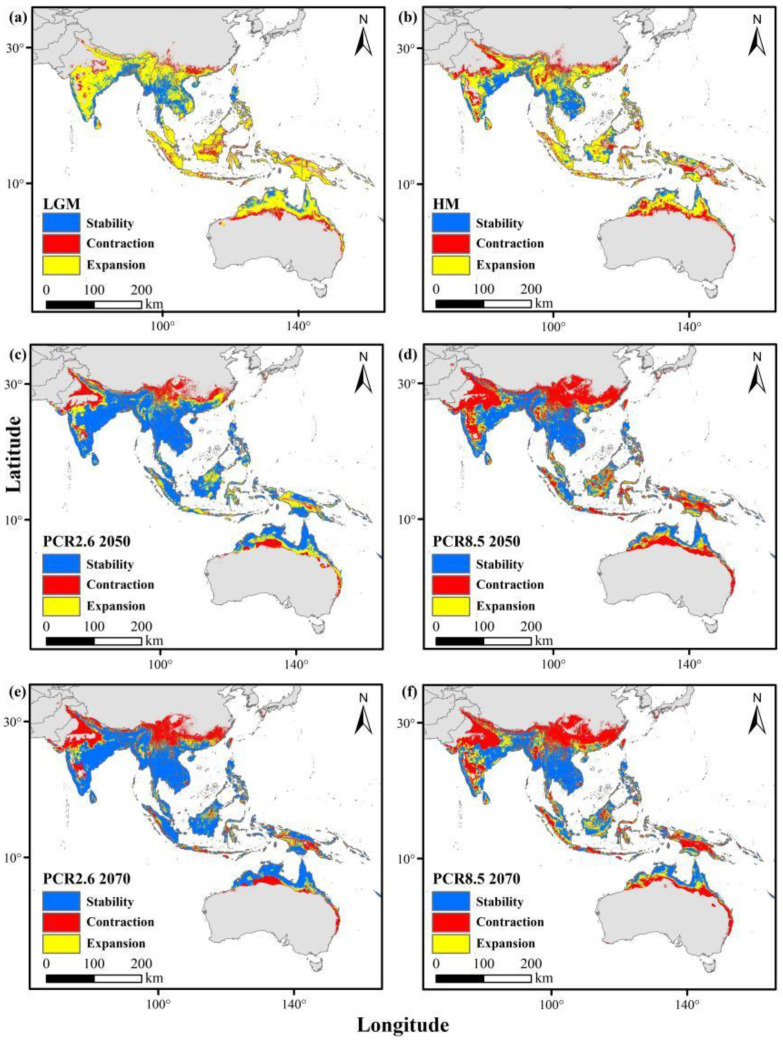
Changes in the distributions of weaver ants from past to current (red areas indicate areas previously suitable and lost by the present period), and from current to future (red areas indicate areas currently suitable which will be lost). (**a**) Last glacial maximum (LGM); (**b**) mid-Holocene (HM); (**c**) future (RCP2.6 2050); (**d**) future (RCP2.6 2070); (**e**) future (RCP8.5 2050); (**f**) future (RCP8.5 2070).

**Figure 5:**
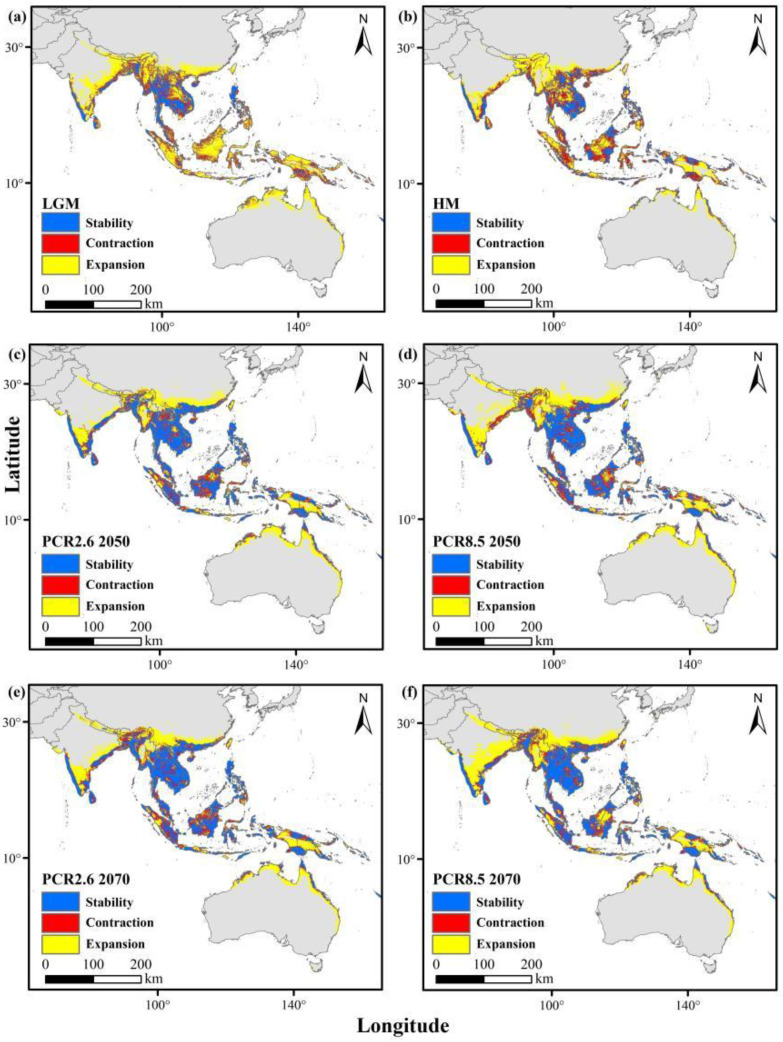
Changes in the distributions of yellow crazy ants from past to current (red areas indicate areas previously suitable and lost by the present period), and from current to future (red areas indicate areas currently suitable which will be lost). (**a**) Last glacial maximum (LGM); (**b**) mid-Holocene (HM); (**c**) future (RCP2.6 2050); (**d**) future (RCP2.6 2070); (**e**) future (RCP8.5 2050); (**f**) future (RCP8.5 2070).

During the last Quaternary ice age and mid-Holocene, their historical ranges were more extensive than they are today. Under the RCP2.6 and RCP8.5 scenarios by 2050 and 2070, distributions in southern China, South and Southeast Asia, and Australia are projected to remain relatively stable. However, weaver ants are anticipated to experience habitat contraction due to climate change (Figure 4), while yellow crazy ants are expected to expand their range (Figure 5).

**Figure 6:**
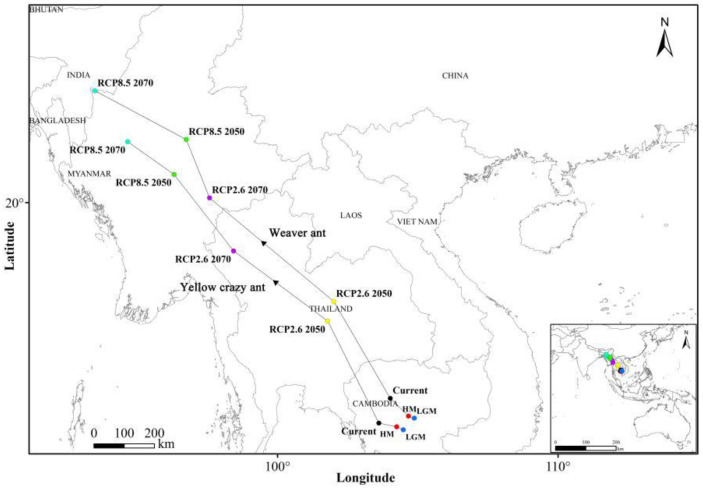
Geometric centroid change of weaver ants and yellow crazy ants in sympatric areas under climate change. Last glacial maximum (LGM); mid-Holocene (HM); future (RCP2.6 2050); future (RCP2.6 2070); future (RCP8.5 2050); future (RCP8.5 2070).

The spatial statistics tool in ArcGIS version 10.8 was employed to calculate the geometric centroids of suitable habitats for both species across various periods. The results indicate that during the last glacial maximum (LGM), the suitable habitats for both ant species shifted northwestward relative to the mid-Holocene (MH) period. In past periods, there were slight biases toward the Malay Archipelago and the Indo-China Peninsula compared to current conditions. Under RCP2.6 scenarios for 2050 and 2070, the suitable habitats continued to migrate northwestward, with a bias toward the Indo-China Peninsula relative to present-day conditions. However, under RCP8.5 scenarios for 2050 and 2070, the suitable habitats shifted toward the Indian Peninsula (Figure 6).

### 3.6. Range Overlap Dynamics of Both Species

The mean values of Schoener’s D (*D*) and Hellinger’s distance I (*I*) were calculated using the ENMTools R package version 1.1.1, resulting in values of 0.74 and 0.92, respectively. These results suggest a substantial degree of spatial niche overlap between both species, as presented in Table 5.

**Figure 7:**
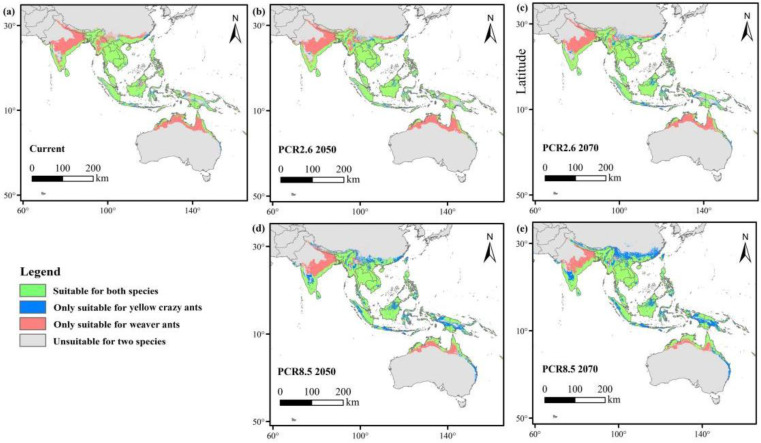
Overlapping maps for weaver ants and yellow crazy ants in the current and future periods. Gray refers to unsuitable areas for both species; blue refers to areas only suitable for yellow crazy ants; pink refers to areas only suitable for weaver ants; green refers to areas suitable for both species. (**a**) Current; (**b**) future (RCP2.6 2050); (**c**) future (RCP2.6 2070); (**d**) future (RCP8.5 2050); (**e**) future (RCP8.5 2070).

The overlapping layers for both species were generated using the overlay analysis tool in ArcGIS 10.8 for both current and future periods. The results revealed that climate change resulted in a contraction of suitable habitat for weaver ants, whereas the suitable habitat for yellow crazy ants expanded. Specifically, under the RCP2.6 and RCP8.5 scenarios in 2050 and 2070, the overlapping area between the two ant species decreased substantially (Figure 7).

The overlap area between the two species was greater under the RCP2.6 scenario but decreased under the RCP8.5 scenario. For yellow crazy ants, suitable habitats initially expanded and subsequently contracted; however, the overall habitat suitability increased under both RCP2.6 and RCP8.5 scenarios. In contrast, suitable habitats for weaver ants progressively diminished as a result of climate change (Table 6).

## 4. Discussion

In response to the growing global challenge of biological invasions, research has increasingly focused on understanding the impacts of invasive species on native ecosystems [1,59]. Our study used field observation, controlled experiments, and species distribution modeling to compare the competitive abilities and projected distribution shifts of the yellow crazy ants and the weaver ants, which exhibit significant geographic overlap. The results show that yellow crazy ants outperformed weaver ants in foraging efficiency and worker mobilization, giving them a significant advantage in acquiring and competing for food resources. This may be due to their faster crawling speed or more effective communication. Research indicates that yellow crazy ants can forage continuously day and night due to their exceptional sustained locomotion ability [4,60]. Previous studies suggest that the invasion success of *Solenopsis invicta* may be attributed to its efficient resource utilization, characterized by rapid food discovery and worker recruitment [61]. Similarly, we observed comparable behaviors in the yellow crazy ant. This phenomenon may be associated with its unique reproductive system, which may facilitate the development of mechanisms for escaping adverse environments or acquiring strong competitive abilities for resources [22]. However, there is currently a lack of relevant evidence to support this assumption. Therefore, investigating the reproductive traits and behavior of yellow crazy ants represents a significant research direction.

Previous research has demonstrated that in ecological competition between two ant species, numerical dominance frequently dictates the outcome of interspecific competition, especially when contesting for food resources in natural habitats [12]. Besides food resource competition, aggression ability is another critical component of competitive capacity for ants. Observations indicate that when a single yellow crazy ant and a single weaver ant coexist, the intensity of their interactions is relatively low, yet both species exhibit comparable aggression abilities. This suggests that native ant species may mitigate the impact of invasive species, potentially limiting their invasion success or rate [62]. As the number of ants increases, both the frequency and intensity of encounters, and mutual attacks between yellow crazy ants and weaver ants escalate. Based on the observed mortality rates, the combat effectiveness of these two species appears to be comparable. Considering that yellow crazy ants are approximately one-third the body length of weaver ants, this suggests that yellow crazy ants may exhibit either disproportionately strong individual combat capabilities or superior intraspecific cooperative behavior. As a dominant ant species in Xishuangbanna, weaver ants exhibit a more diverse diet and possess stronger ecological competitiveness compared to other native ant species [40]. This implies that yellow crazy ants may pose a greater threat to smaller or less aggressive native ant species. In addition to resource competition and combat ability, the tolerance of ants to harsh environments also significantly influences their survival and distribution.

Our results also demonstrated that weaver ants can survive for up to 96 h without food or water, whereas yellow crazy ants can endure for up to 120 h under the same conditions. In artificially induced extreme conditions, the lack of coordination and interspecies cooperation may significantly reduce survival time. In natural habitats, yellow crazy ants may exhibit longer survival times compared to laboratory settings. Despite their smaller size, this invasive species displays greater resilience to starvation and dehydration, thereby enhancing their tolerance to harsh environments. This study substantiates the environmental adaptability and ecological competitiveness of yellow crazy ants through the comprehensive analyses of resource competition, combat behavior, and tolerance to extreme environments. Chimeras may lead to yellow crazy ants having a stronger ability to adapt to the environment and establish new colonies. Our experimental results provide certain evidence from an ecological perspective [22]. Unlike much of the existing research on ant competition, which has predominantly relied on qualitative assessments [63], our study introduces a novel quantitative framework for analyzing interspecies competition among ants.

As a representative insect group, the distribution of ants is influenced not only by local competitors, but also by various abiotic environmental factors [26]. This study investigates the competitive interactions between two ant species and assesses the potential impacts of global climate change on their overlapping area dynamics [64]. By employing species distribution modeling, we simulated and projected changes in the distribution of two ant species under historical, current, and future scenarios. The results demonstrate that the most influential factors on their distribution are annual average temperature, monthly average diurnal temperature range, annual temperature range, isothermality, and rainfall in the wettest month. This indicates that climatic factors are the predominant determinants shaping the large-scale distribution patterns of these ant species [65]. However, at a local scale, biological factors and microhabitat conditions can also significantly influence species distribution. The adaptation of ant species to both biotic and abiotic environments collectively determines their distribution patterns [26,66]. The simulation results, which account for environmental factors, suggest that the potential suitable habitats for the two ant species are primarily concentrated in the Indian Peninsula, Indochinese Peninsula, and Malay Archipelago, all regions characterized by tropical monsoon and tropical rainforest climates. These findings are consistent with existing data records. The suitable habitats of these ant species have historically exhibited a pattern of initial expansion followed by subsequent contraction, with the primary range of distribution change concentrated in southern China, South Asia, Southeast Asia, and northern Australia. From the present to the future, two ant species distributed from southern China to north Malaysia are projected to maintain relatively stable suitable habitats. The temperature increase resulting from global climate change deferentially impacts the future suitable habitats of these two ant species: the suitable habitat for weaver ants is generally constricting, whereas that for yellow crazy ants, it is expanding. Specifically, the constriction in the suitable habitat for weaver ants is most pronounced in the Indian Peninsula and the southern regions of the Malay Archipelago. This trend can likely be attributed to the relatively weaker adaptability of weaver ants to temperature changes compared to yellow crazy ants [67].

The potential distribution area prediction indicates that the two ant species exhibit a significant overlap in suitable habitats under current climatic conditions, primarily concentrated in the Indochinese Peninsula, southern India, and parts of the Malay Archipelago. Under future climate scenarios RCP2.6 and RCP8.5 in 2050 and 2070, this overlap is projected to diminish. According to the niche theory, closely related species coexisting in the same area are likely to compete for essential resources such as food, water, and space. As a result, the dominant species may expand its ecological niche through competitive mechanisms like aggression or competitive exclusion [68,69]. By integrating insights from food resource competition experiments with species distribution models, we can predict that the suitable habitats and overlapping areas of the two ant species may undergo significant changes in the future. Current models predominantly focus on abiotic factors such as environmental and climatic conditions, which can simulate species distribution and changes to some extent. However, these models may not fully capture the complexities of species distribution dynamics. We hypothesize that incorporating biotic factors, including resource competition and ecological adaptability, into model parameters could reveal that the actual expansion rate of invasive species surpasses current simulation outcomes. Due to current technological limitations, integrating all relevant factors into species distribution models for precise analysis remains a significant challenge. Our research projects that the habitat suitable for yellow crazy ants will expand, while the overlap with weaver ants’ habitat will diminish. This shift may weaken the regulatory effect of weaver ants on yellow crazy ant populations over time. Furthermore, analogous trends are likely to be observed in other native species that currently inhibit the expansion of the yellow crazy ant, potentially resulting in a synergistic effect between the decline of native species and the impacts of climate change. This could heighten the probability of yellow crazy ant range expansion and cause substantial global invasive damage [17,70,71]. However, it remains uncertain whether this will lead to an invasion on par with that of the red fire ant. Considering its distinctive genetic traits, documented adverse invasive effects, and our research findings, we strongly advocate for enhanced monitoring and further research to mitigate the risk of a severe invasion event [72].

Predicting the potential distribution areas of invasive species is a crucial step in the prevention and control of biological invasions [1]. A key component in understanding the distribution dynamics of invasive species involves examining the interactions between invasive and native species. However, research in this area—both qualitative and quantitative—remains limited. As a pivotal driver of biodiversity changes, biological invasions will remain a critical focus for conservation biologists [73]. Despite the importance of this issue, research on the interactions between invasive and native species remains notably limited, with both qualitative and quantitative studies in invaded areas being insufficient. Most species only receive investigation and management efforts following severe invasions, which significantly increases the challenges of prevention and control. This delayed response also leads to irreversible impacts on biodiversity and ecosystems. We conducted an analysis of the potential development trends of two ant species by simulating ecological niche competition and modeling suitable habitats. Our study adopted an integrative and multidimensional approach, demonstrating that enhanced surveillance of the yellow crazy ant is crucial for mitigating potential invasive risks. This research not only addresses certain limitations identified in previous studies, but also offers valuable insights for future investigations into species distribution dynamics.

## 5. Conclusions

The invasive behavior of yellow crazy ants may confer direct competitive advantages over weaver ants through aggressive interactions, predation, and competition for habitat and resources. Furthermore, under scenarios of global climate change characterized by high temperatures and drought, Southeast Asia is likely to experience as much drought as flooding, and extreme weathers are expected. Yellow crazy ants have demonstrated adaptive habitat expansion, whereas weaver ants experience constraints. The effective long-term monitoring of yellow crazy ant invasions and addressing climate change are crucial for maintaining the ecological balance for weaver ants. Our study reveals that the overlapping area between the two ant species is diminishing due to the relatively weaker adaptability of weaver ants to temperature changes compared to yellow crazy ants. Overall, our research underscores interspecies interactions, and provides insights into the environmental factors influencing the invasion dynamics of yellow crazy ants and their impact on weaver ants in Southeast Asia. Climate change is projected to substantially alter the distribution patterns of both invasive and native species at a global scale. Consequently, it is imperative to investigate the impacts of climate change on species interactions and distribution dynamics in greater depth.

## Figures and Tables

**Table 1:** Environment variables utilized in the construction of the species distribution model.

Category	Code	Environmental Variable
Climate variable	bio1	Annual Mean Temperature
	bio2	Monthly Average Diurnal Temperature Range
	bio3	Isothermality (bio2/bio7) (×100)
	bio7	Annual Temperature Variation (bio5-bio6)
	bio13	Precipitation of the Wettest Month
	bio14	Precipitation of the Driest Month
	bio15	Precipitation Seasonality (Coeffificient of Variation)
	bio18	Warmest Season Precipitation
	bio19	Coldest Season Precipitation
Terrain variable	alt	Altitude
	sl	Slope
	asp	Aspect

**Table 2:** Comparison of bait detection time and the maximum number of recruited worker ants between yellow crazy ants and weaver ants across different food sources.

Bait	Bait Detection Time (min)		Maximum Number of Recruited Workers	
	Yellow Crazy Ants	Weaver Ants	Yellow Crazy Ants	Weaver Ants
Apple	3.26 ± 0.43 A	21.50 ± 1.92 B	20.47 ± 3.34 a	1.73 ± 0.25 b
Honey	4.19 ± 0.58 A	10.21 ± 1.11 B	86.20 ± 7.59 a	3.40 ± 0.72 b
Sausage	7.25 ± 1.62 A	8.37 ± 1.53 A	12.07 ± 3.13 a	49.67 ± 6.78 b

Note: Data in the table are the mean ± S.E. Different capital letters in the bait detection time row indicate significant differences between the two species, while different lowercase letters in the maximum number of recruited workers row indicate significant the differences between the two species (*p* < 0.01, *t*-test).

**Table 3:** Contribution rate and importance affecting the range of both species.

Environmental Variable Monthly Average Diurnal Temperature Range	Code	Yellow Crazy Ants		Weaver Ants	
		Contribution (%)	Importance (%)	Contribution (%)	Importance (%)
Annual Mean Temperature	bio1	**14.8**	**26.1**	**15.1**	**37.1**
Monthly Average Diurnal Temperature Range	bio2	**15**	**32.2**	**5.3**	**10.5**
Isothermality	bio3	2.2	2.2	**6.9**	**10.2**
Annual temperature variation	bio7	**29.7**	**8.1**	4.2	4.9
Precipitation of Wettest Month	bio13	**34.6**	**17.1**	**63.4**	**23.8**
Precipitation of Driest Month	bio14	0.2	0.9	0.2	0.4
Precipitation Seasonality (Coeffificient of Variation)	bio15	0.7	1.6	0.3	2.4
Warmest Season Precipitation	bio18	0.6	3.2	1.1	3
Coldest Season Precipitation	bio19	0.5	2.9	1.3	3.1
Altitude	alt	0.3	1.7	1.2	2.7
Slope	sl	0.9	2.1	0.9	1.7
Aspect	asp	0.5	1.9	0.1	0.2

Note: Variables with more than 5% contribution and importance are highlighted in **bold**.

**Table 4:** The optimal climatic ranges of both species.

Environmental Variable	Code	Yellow Crazy Ants		Weaver Ants	
		Suitable Ranges	Most Suitable Value	Suitable Ranges	Most Suitable Value
Annual Mean Temperature (°C)	bio1	13.52–30.79	29.34	17.83–30.78	27.65
Mean diurnal range (Mean of monthly (max temp-min temp)) (°C)	bio2	1.69–20.67	8.16	1.84–20.02	8.09
Isothermality (bio2/bio7) (×100) (%)	bio3	9.61–100	59.45	9.55–100	50.96
Temperature annual range (bio5-bio6) (°C)	bio7	3.57–32.34	9.24	3.67–25.08	9.05
Precipitation of wettest month (mm)	bio13	<2062	1686.03	<2059	1934.36

**Table 5:** Niche overlap index (*D*) and (*I*) values of weaver ants and yellow crazy ants in different epochs.

Epoch	Niche Overlap INDEX	
	Schoener Value (*D*)	Hellinger-Based Value (*I*)
Current epoch	0.763	0.936
RCP2.6 2050	0.740	0.921
RCP2.6 2070	0.746	0.927
RCP8.5 2050	0.736	0.919
RCP8.5 2070	0.726	0.914

**Table 6:** The percentage of suitable habitats in the study area for both species in different periods.

Species	Area (%)						
	LGM	MH	CE	RCP2.6 2050	RCP2.6 2070	RCP8.5 2050	RCP8.5 2070
Yellow crazy ants	14.3	11.4	5.6	7.6	8.2	11.4	12.4
Weaver ants	17.9	14.2	7.4	6.8	6.3	5.8	5.4
Overlap	N.D.	N.D.	3.05	2.89	2.99	2.73	2.67

Note: Last glacial maximum (LGM); mid-Holocene (HM); current epoch (CE); future (RCP2.6 2050); future (RCP2.6 2070); future (RCP8.5 2050); future (RCP8.5 2070); not detected (N.D.).

## Data Availability

The data that support the findings of this study are available from the corresponding author upon reasonable request.
